# Of Mice and ‘Convicts’: Origin of the Australian House Mouse, *Mus musculus*

**DOI:** 10.1371/journal.pone.0028622

**Published:** 2011-12-12

**Authors:** Sofia I. Gabriel, Mark I. Stevens, Maria da Luz Mathias, Jeremy B. Searle

**Affiliations:** 1 Department of Biology, University of York, York, United Kingdom; 2 CESAM–Centre for Environmental and Marine Studies, Departamento de Biologia Animal, Faculdade de Ciências da Universidade de Lisboa, Lisbon, Portugal; 3 South Australian Museum, and School of Earth and Environmental Sciences, University of Adelaide, Adelaide, Australia; 4 Department of Ecology and Evolutionary Biology, Cornell University, Corson Hall, Ithaca, New York, United States of America; University of Akron, United States of America

## Abstract

The house mouse, *Mus musculus*, is one of the most ubiquitous invasive species worldwide and in Australia is particularly common and widespread, but where it originally came from is still unknown. Here we investigated this origin through a phylogeographic analysis of mitochondrial DNA sequences (D-loop) comparing mouse populations from Australia with those from the likely regional source area in Western Europe. Our results agree with human historical associations, showing a strong link between Australia and the British Isles. This outcome is of intrinsic and applied interest and helps to validate the colonization history of mice as a proxy for human settlement history.

## Introduction

Invasive species have a major impact on Australia, threatening native biodiversity and causing massive costs to agriculture every year. The house mouse (*Mus musculus*) is a highly successful invader worldwide and particularly throughout mainland Australia and surrounding islands [1], but the source of this invasion is still unknown. Over the last century the house mouse has become a serious agricultural pest in Australia, particularly in grain growing regions where it shows aperiodic but increasingly frequent outbreaks [2], [3]. This ability to respond rapidly to favorable environmental conditions, reaching plague proportions over large areas, is the most striking trait that distinguishes Australian house mice from their conspecifics around the world [2]. However, no attempt has yet been made to determine the geographical origin(s) of the first mouse colonists. Australia was probably the last continental landmass to be colonized by the house mouse, presumably remaining mouse-free until the arrival and settlement of the first Europeans colonists, about two centuries ago [2]. The earliest Australian specimen registered at a museum was collected in Tasmania in 1884 (Cat. No. M106, Australian Museum). The arrival of the British First Fleet in 1788 is often cited as the most probable origin of house mice into Australia but earlier introductions were possible with Dutch ships charting the former “New Holland” coast since the early 1600 s. To determine the source area of Australian mice we have followed a phylogeographic approach involving the analysis of mitochondrial D-loop sequences, as previously adopted elsewhere in Australasia when New Zealand and nearby islands were analyzed [4]. D-loop sequences are the only molecular marker for which there are substantial data throughout the distribution of the house mouse and mitochondrial DNA appears to be particularly useful as a phylogeographic tool to elucidate initial colonization events in this species [5]. In our study, we compare the haplotypes of mice from Australia with available haplotypes of mice from likely geographical source areas in Western Europe, including published sequences from the British Isles [6], [7] and new sequences from the Netherlands.

## Results

**Figure 1:**
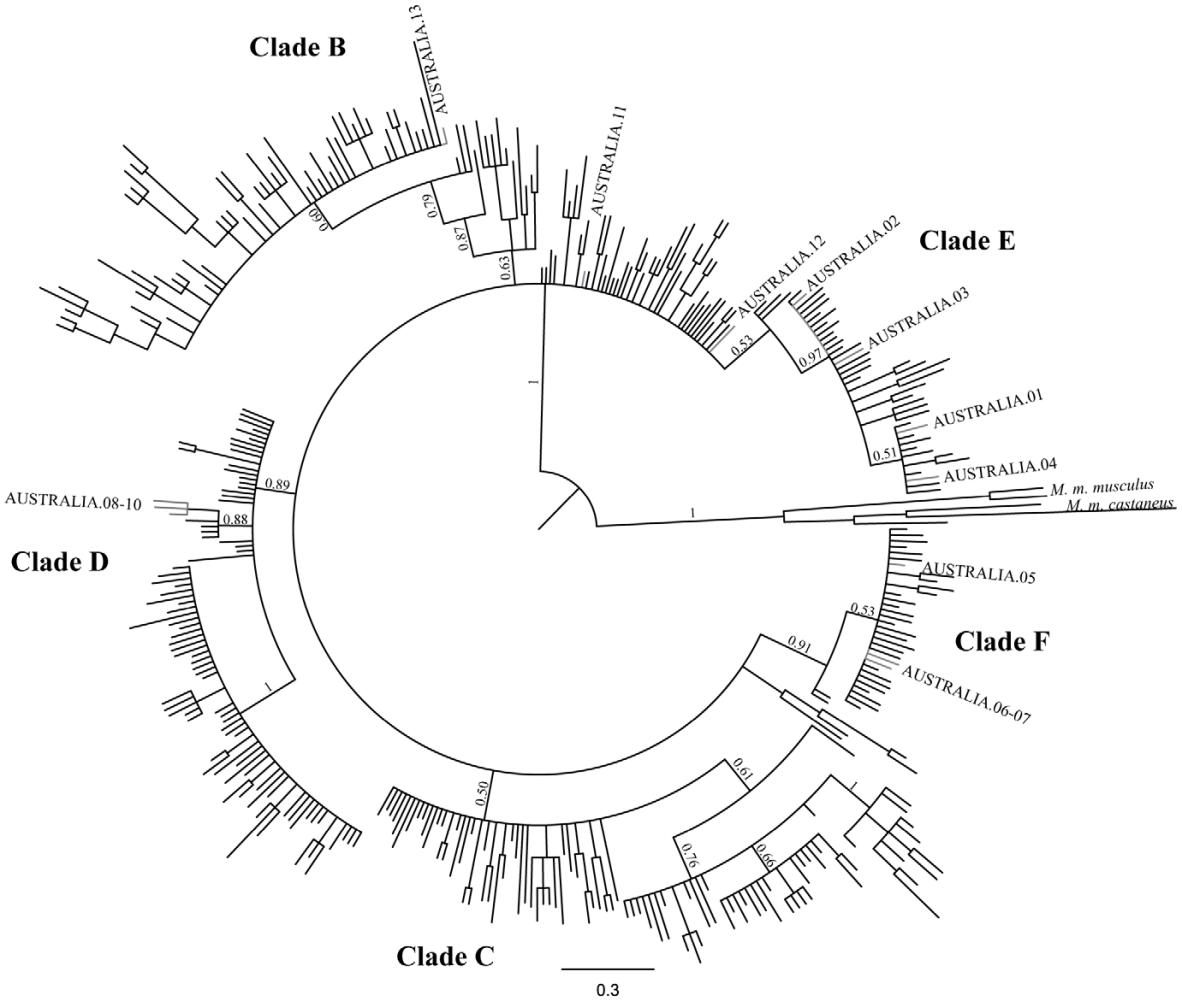
Phylogenetic tree for *Mus musculus domesticus* obtained after Bayesian analysis based on D-loop sequences (published and this study). Posterior probabilities ≥0.50 are shown at the nodes of each clade, labeled according to [7]. Australian haplotypes are highlighted in the tree (see Figure S1 for further details).

**Figure 2:**
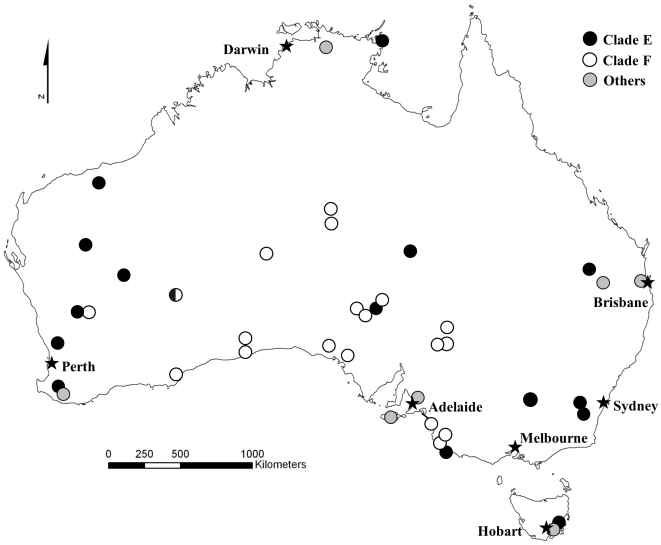
House mouse collection localities in mainland Australia, Kangaroo Island and Tasmania. Circles correspond to mtDNA haplotypes grouped by lineage. One locality was characterized by mice of both clade E and clade F.

All new sequences examined (Australian and Dutch) were typical of the western European house mouse *Mus musculus domesticus* rather than one of the other subspecies (*musculus*, *castaneus*, *gentilulus* [8]–[10]) and therefore our phylogenetic tree was based purely on *domesticus* haplotypes and clades were labeled according to previous practice for this subspecies (Figures 1 and S1) [7]. The sequences from the potential source area of the Netherlands were largely clade E, which is also predominant in Britain and northern France (Figure S1). The Australian sequences harbored 13 distinct haplotypes belonging to four clades (Figure 1). The majority of sequences were evenly divided amongst two clades: E (*N* = 32; haplotypes AUSTRALIA.01–04) and F (*N* = 27; AUSTRALIA.05–07), each with a wide geographic distribution (Figure 2). Of the sequences assigned to clade E, 81% belonged to haplotype AUSTRALIA.01, previously described as U47431 found in the British Isles [6]–[8], [11], France [7], Netherlands (this study, NETHERLANDS.02, *N* = 1), Germany [8], Denmark [8], Norway [9], [12], Cameroon [13], New Zealand [4] and the sub-Antarctic Kerguelen Archipelago [14]. Likewise, 93% of the clade F sequences belonged to haplotype AUSTRALIA.05, previously described as U47495 and also found in Scotland [7], Croatia [9] and New Zealand [4]. The remaining sequences corresponded to new haplotypes, distributed among clade D (*N* = 9; AUSTRALIA.08–10), restricted to a small area in Australia's east coast, clade B (*N* = 1; AUSTRALIA.13) and eight sequences basal within the tree (AUSTRALIA.11–12), largely from Kangaroo Island and Tasmania (Figure 2). In addition to AUSTRALIA.01 and 05, only two other Australian haplotypes have previously been described elsewhere: AUSTRALIA.02 (*N* = 4; mainland Australia) previously recorded as U47432 in Scotland [8], Germany [8], France [7], [14], the Netherlands (this study, NETHERLANDS.03, *N* = 2) and New Zealand [4], and AUSTRALIA.11 (*N* = 6; Kangaroo Island and adjacent coast) previously recorded as Turkey.7 in Istanbul [15].

Given that the predominant clades in Australia, E and F, are also found in the potential source areas of the Netherlands, Britain and Ireland, Table 1 compares nucleotide and haplotype diversity between these four regions plus New Zealand, as another colonized area in Australasia with a similar settlement history. The haplotype diversity is higher in the potential source areas, particularly on a clade-by-clade basis. The overall nucleotide diversity is not systematically higher in the source areas, but Australia in particular has much lower nucleotide diversities than the potential source areas within separate clades.

**Table 1:** Genetic diversity indices applied to house mice with *Mus musculus domesticus* D-loop sequences by country and by prevalent mtDNA clade (E and F).

Country/		Number of			
clade	*N*	haplotypes	*h*	*π*	Reference
**Britain**					
Overall	89	31	0.932±0.013	0.0087±0.0045	
Clade E	37	9	0.757±0.047	0.0033±0.0020	[7]
Clade F	45	18	0.900±0.030	0.0033±0.0020	
**Ireland**					
Overall	81	19	0.887±0.017	0.0057±0.0031	
Clade E	20	5	0.663±0.069	0.0016±0.0011	[7]
Clade F	60	13	0.830±0.027	0.0019±0.0013	
**Netherlands**					
Overall	17	7	0.831±0.065	0.0109±0.0059	
Clade E	11	4	0.691±0.128	0.0023±0.0016	This study
Clade F	-	-	-	-	
**Australia**					
Overall	77	13	0.772±0.031	0.0072±0.0003	
Clade E	32	4	0.333±0.100	0.0007±0.0002	This study
Clade F	27	3	0.145±0.090	0.0002±0.0001	
**New Zealand**					
Overall	79	10	0.675±0.049	0.0059±0.0032	
Clade E	60	5	0.454±0.060	0.0015±0.0011	[4]
Clade F	6	2	0.333±0.215	0.0004±0.0005	

***N*** – number of individuals analyzed.

***h*** – haplotype diversity.

***π*** – nucleotide diversity.

## Discussion

### Colonization of Australia by house mice

All Australian D-loop sequences examined belonged to the subspecies *Mus musculus domesticus*. This fits with the taxonomic status based on morphology [16], [17] and lymphocyte antigens [18]. This does not mean that other subspecies are not present. Some of our samples were deliberately chosen as *domesticus*, but in most cases the specimens we used were classified as ‘*Mus musculus*’ with no attempt to define subspecies, yet all were characterized as *domesticus* on D-loop sequencing. Thus, from our wide-ranging studies, it appears that *M. m. domesticus* is the predominant subspecies of house mouse in Australia. This accords well with the hypothesis tested here that house mice arrived from within the range of the western European *domesticus* subspecies [17], either on Dutch or British ships, and not from the closer potential source region (Southeast Asia) where a different subspecies is present, *Mus musculus castaneus* [19].

Looking in fine detail at the D-loop clades and haplotypes present, the specific link with the British Isles becomes clearer. The two most widely distributed clades in Australia are also the two most widely distributed clades in the British Isles: clades E and F. The haplotype of clade E that has been found in most localities in Britain (nine sites in southern Scotland, Wales and southern England [6], [7]) is also the most widespread and frequent haplotype of that clade in Australia (AUSTRALIA.01), but has only been detected in one out of 17 individuals from the Netherlands. Clade F is well documented around the northern and western periphery of the British Isles and the widespread clade F haplotype in Australia (AUSTRALIA.05) has been also found there (in a museum specimen dating from 1914 from the Isle of Lewis off the coast of Scotland [7]). On the other hand, clade F was not present among the Dutch mice we examined, nor has it been detected in the surrounding regions of continental Europe previously sampled in France, Germany and Denmark [7]–[9], [13], except for three specimens out of 43 collected in northern France (taken from a single location in Abbeville [7]). The low frequency of AUSTRALIA.01 ( = NETHERLANDS.02) and absence of clade F in our sample of contemporary Dutch mice does not fully exclude the Netherlands as a possible source area for the Australian house mouse. However, pending larger sample sizes and more detailed genetic data, a British Isles origin of Australian mice is the most reasonable interpretation of our results.

Therefore, our results agree with the historical routes of colonization, further suggesting that house mice were brought to Australia from at least two parts of the British Isles (northwestern Britain and/or Ireland [clade F] and somewhere in southern Britain [clade E]) probably early in the settlement of Australia. The early historical links between Britain and Australia date back to the claiming of Australia as part of the British Empire by James Cook in 1770. Although New Holland had already been discovered and named by the Dutch in 1606 and the west coast visited on a number of occasions, no trade or settlement was attempted at this time. The 11 ships that constituted the British First Fleet arrived in January 1788 at Botany Bay, near the present site of Sydney, to establish the first European penal colony in Australia. During the following years two other convoys also comprising numerous ships carrying settlers and supplies arrived in the newly founded colony of New South Wales. Throughout these early stages of establishment, thousands of English, Scottish and Irish convicts were transported to Australia leaving mainly from southern England and Ireland, bringing provisions and livestock with them [20]. Therefore, it seems reasonable to believe that the first mouse colonists, survivors of the months-long journey, arrived on this occasion, rapidly expanding their range throughout Australia, spreading the genetic signature of their geographic origin – clades E and F. Mice of other clades are restricted to coastal areas of Australia, near major harbors: Perth, Adelaide, Hobart, Brisbane and Darwin (Figure 2) and therefore most probably represent pockets of secondary colonization events. Thus, the genetic signature of the primary colonization appears to be retained in the extant mouse populations with a limited impact of subsequent introductions. This same phylogeographic pattern is found recurrently when house mouse populations from small isolated islands are surveyed [e.g. 4], [14], [21]–[23] but is particularly striking for a landmass as huge as Australia. Although Australia is nearly as large as Europe in area, the dynamics of arrival of house mice is still effectively like that of a small island, i.e. with a limited number of entry points and expansion from those.

The two most common haplotypes in Australia have also been found in New Zealand, with AUSTRALIA.01 being the most widespread and abundant haplotype there (scored as NZ.4) [4]. Given the shared history of activities involving British ships during settlement of the two countries this is not surprising [4], [24]. It is interesting that genetic diversity within mitochondrial clades was higher for New Zealand than for Australia. That may be because New Zealand is a multitude of islands and the sampling reflected that [4]. In particular, among the small islands of the archipelago, the mice tended to differ in D-loop haplotype, increasing overall diversity, and with the inference that the different islands had different colonization histories [4]. The sampling of Australia was dominated by specimens from the mainland where haplotypes AUSTRALIA.01 and AUSTRALIA.05 managed to spread far and wide. The observed low haplotype diversity on the Australian landmass is either the reflection of low diversity among the founders or small numbers of them, or post-colonization population bottlenecking.

Overall then, the mitochondrial data presented here provide valuable information on the colonization history of Australia by the house mouse. There are strong indications that mitochondrial DNA is a particularly useful marker for first colonization [6], [14]. However, the sequence that we used, the D-loop, is only a small part of the mitochondrial genome. More precision on the source and timing of initial colonization of Australia by house mice, associated population sizes, and the detail of secondary colonization events, will be provided by studies involving larger numbers of individuals, complete mitochondrial genome sequencing, and substantive analysis of the nuclear genome [5], [7], [25]. Nuclear genome data has the potential to provide considerably more information than currently available, including the perspective of both male and female colonization (as the maternally inherited mitochondrial genome only reflects female colonization).

### The house mouse as a model organism in invasion biology

Studies on species-invasion have provided invaluable insights into ecological and evolutionary processes, highlighting the utility of alien species as model organisms [26]. In a mammalian context the house mouse is an iconic example of a successful invader [27], capable of thriving in the most remote and inhospitable locations on the planet, particularly on islands. Given that house mice have colonized islands across all climatic and biogeographic regions, island invasions constitute valuable experiments to test for local adaptation to different biotic and abiotic factors. Australia, a continent with characteristics similar to an oceanic island in terms of its invasibility, constitutes one of the most dramatic house mouse invasion zones worldwide. Knowing that Australian house mice likely came from the British Isles allows comparison of populations from the source and colonized areas, with all the genomic tools available for this model species. This could result in a better understanding of characteristics of Australian house mice such as their distinctive predisposition to plagues [2].

It has been claimed that through phylogeographic analysis house mice are good proxies for human movement patterns and settlement history over the last few thousand years because of their remarkable tendency to be transported wherever humans go [27]. The close match between post-European human settlement history and house mouse phylogeography in Australia supports this contention. It is noteworthy that house mice did not colonize from the closest geographical region, Southeast Asia, even though house mice (*castaneus* subspecies) have long been present there [19]. There is evidence of a long pre-European trade period between Australia, Indonesia and the Asian mainland, perhaps three to four centuries prior to the arrival of the first Europeans [28]. Apparently, despite this early nautical and commercial activity, propagule pressure of *castaneus* mice was not sufficient to allow them to invade and spread throughout Australia. It appears that only with the arrival and establishment of the British colony the invasion risk became significantly high, allowing the successful introduction of mice.

## Materials and Methods

### Samples

We obtained a total of 77 house mouse tissue and DNA samples representing 38 locations throughout mainland Australia, one from Kangaroo Island and six from Tasmania (Figure 2). The samples were provided by museums and private collectors (see Table S1 for details).

During the last two decades hundreds of D-loop sequences of *Mus musculus domesticus* have been published from locations all over Western Europe, including the British Isles, and our analysis includes all those available at the time (Figure S1). However, there were no sequences from the Netherlands and therefore, considering its potential as a source area for Australia, an additional set of 17 tissue samples from 10 Dutch localities were obtained from the Zoological Museum of Amsterdam and sequenced (Table S1).

### Molecular methods

Genomic DNA was extracted from all samples using the Qiagen DNeasy Blood & Tissue extraction Kit according to the manufacturer's guidelines. For the Australian samples, complete mitochondrial D-loop and flanking regions were amplified by PCR with the primer pair L15774 - 5′-TGAATTGGAGGACAACCAGT-3′ and H2228 - 5′-TTATAAGGCCAGGACCAAAC-3′, using standard concentrations of DNA and reagents as described in [6]. The DNA recovered from the Netherlands museum samples was degraded and was amplified in three overlapping fragments with the following primer pairs (designed here): dloopF1-5′-GCACCCAAAGCTGGTATTCT-3′ and dloopR1-5′-TTGTTGGTTTCACGGAGGAT-3′, dloopF2 - 5′-ACTATCCCCTTCCCCATTTG - 3′ and dloopR2 - 5′-GATTGGGTTTTGCGGACTAA-3′, dloopF3-5′-ATAGCCGTCAAGGCATGAAA-3′ and dloopR3-5′-GCTTTGCTTTGTTATTAAGCTACA-3′.

### Data analysis

All haplotypes that were obtained are deposited in GenBank (JF277281-JF277300). These were combined with 367 previously published haplotypes of *Mus musculus domesticus* for phylogenetic analysis (Figure S1). For the broadest possible comparison, the sequences were truncated to a standard length of 853 base pairs, including the whole D-loop. Based on the DNA substitution model retrieved from jModelTest0.1.1 [29], the dataset was analyzed using the TIM2+I+Γ model for Bayesian MCMC inference [30] through two simultaneous runs of five chains each, during 10 million generations sampled every 1000 steps. After a 30% burn-in, both runs were used to generate a 50% majority rule consensus tree. *Mus musculus musculus* and *M. m. castaneus* sequences were used as outgroups. In comparisons involving Australian mice, haplotype (*h*) and nucleotide (*π*) diversities were determined using Arlequin 3.11 [31].

## Supporting Information

Figure S1 **Detailed phylogenetic tree of** ***Mus musculus domesticus*** **.** This is the detailed 50% majority rule consensus tree after Bayesian inference that is summarized in Figure 1 in the paper (based on a total of 378 *Mus musculus domesticus* D-loop haplotypes, derived from this study and all previous data available at the time of analysis) (Prager *et al.* 1993, 1996, 1998; Nachman *et al.* 1994; Gündüz *et al.* 2000, 2001, 2005; Ihle *et al.* 2006; Rajabi-Maham *et al.* 2008; Förster *et al.* 2009; Searle *et al.* 2009a, b; Jones *et al.* 2011). Posterior probabilities of 0.50 and above are shown on branch nodes. All haplotypes detected in this study are highlighted with an asterisk in the tree (details of samples can be found in Table S1). Codes indicate GenBank number or code used in the publication where the sequence was first reported, followed by a list of all the countries (country codes) where the haplotype has been recorded: AR, Argentina; AU, Australia, BG, Bulgaria; CH, Switzerland; CM, Cameroon; CY, Cyprus; DE, Germany; DK, Denmark; EG, Egypt; ES, Spain; (ES), Canary Islands (Spanish dependency); FI, Finland; FR, France; GB, Great Britain; GE, Georgia; GR, Greece; HR, Croatia; IE, Ireland; IL, Israel; IR, Iran; IT, Italy; LU, Luxembourg; MA, Morocco; MR, Mauritania; NE, Niger; NO, Norway; NL, Netherlands; NZ, New Zealand; PE, Peru; PT, Portugal; (PT), Madeiran Archipelago (Portuguese dependency); SE, Sweden; TR, Turkey; US, United States.
(PDF)

Table S1 **Details of all house mice obtained from Australia and the Netherlands subject to D-loop sequencing.** Geographical coordinates are represented as decimal degrees. Most Australian samples were provided by Museum collections and the remaining samples belong to private collections of Michael Nachman, Kristin Ardlie [Ardlie KG, Silver LM (1998) Low frequency of t haplotypes in natural populations of house mice (*Mus musculus domesticus*). Evolution, *52*, 1185–1196] and Michael Driessen. Samples provided by Michael Driessen were collected during pest management work of the Resource Management and Conservation Division, Department of Primary Industries, and Water, Tasmania, following their standard ethical practice. All Dutch samples were provided by Adri Rol at the Zoological Museum of Amsterdam. ‘Sample ID’ corresponds to the original Museum Catalogue Number.
(PDF)
