# Supplementary material for: Of Mice and ‘Convicts’: Origin of the Australian House Mouse, Mus musculus
Source: PLoS One. 2011 Dec 12;6(12):e28622. doi: 10.1371/journal.pone.0028622 (PMC3236204; doi:10.1371/journal.pone.0028622)
Supplement: Figure S1 — Detailed phylogenetic tree of Mus musculus domesticus . This is the detailed 50% majority rule consensus tree after Bayesian inference that is summarized in Figure 1 in the paper (based on a total of 378 Mus musculus domesticus D-loop haplotypes, derived from this study and all previous data available at the time of analysis) (Prager et al. 1993, 1996, 1998; Nachman et al. 1994; Gündüz et al. 2000, 2001, 2005; Ihle et al. 2006; Rajabi-Maham et al. 2008; Förster et al. 2009; Searle et al. 2009a, b; Jones et al. 2011). Posterior probabilities of 0.50 and above are shown on branch nodes. All haplotypes detected in this study are highlighted with an asterisk in the tree (details of samples can be found in Table S1). Codes indicate GenBank number or code used in the publication where the sequence was first reported, followed by a list of all the countries (country codes) where the haplotype has been recorded: AR, Argentina; AU, Australia, BG, Bulgaria; CH, Switzerland; CM, Cameroon; CY, Cyprus; DE, Germany; DK, Denmark; EG, Egypt; ES, Spain; (ES), Canary Islands (Spanish dependency); FI, Finland; FR, France; GB, Great Britain; GE, Georgia; GR, Greece; HR, Croatia; IE, Ireland; IL, Israel; IR, Iran; IT, Italy; LU, Luxembourg; MA, Morocco; MR, Mauritania; NE, Niger; NO, Norway; NL, Netherlands; NZ, New Zealand; PE, Peru; PT, Portugal; (PT), Madeiran Archipelago (Portuguese dependency); SE, Sweden; TR, Turkey; US, United States. (PDF) [file pone.0028622.s001.pdf]

Figure S1 Detailed phylogenetic tree of *Mus musculus domesticus*. This is the detailed 50% majority rule consensus tree after Bayesian inference that is summarized in Figure 1 in the paper (based on a total of 378 *Mus musculus domesticus* D-loop haplotypes, derived from this study and all previous data available at the time of analysis) (Prager *et al.* 1993, 1996, 1998; Nachman *et al.* 1994; Gündüz *et al.* 2000, 2001, 2005; Ihle *et al.* 2006; Rajabi-Maham *et al.* 2008; Förster *et al.* 2009; Searle *et al.* 2009a, b; Jones *et al.* 2011). Posterior probabilities of 0.50 and above are shown on branch nodes. All haplotypes detected in this study are highlighted with an asterisk in the tree (details of samples can be found in Table S1). Codes indicate GenBank number or code used in the publication where the sequence was first reported, followed by a list of all the countries (country codes) where the haplotype has been recorded: AR, Argentina; AU, Australia, BG, Bulgaria; CH, Switzerland; CM, Cameroon; CY, Cyprus; DE, Germany; DK, Denmark; EG, Egypt; ES, Spain; (ES), Canary Islands (Spanish dependency); FI, Finland; FR, France; GB, Great Britain; GE, Georgia; GR, Greece; HR, Croatia; IE, Ireland; IL, Israel; IR, Iran; IT, Italy; LU, Luxembourg; MA, Morocco; MR, Mauritania; NE, Niger; NO, Norway; NL, Netherlands; NZ, New Zealand; PE, Peru; PT, Portugal; (PT), Madeiran Archipelago (Portuguese dependency); SE, Sweden; TR, Turkey; US, United States.

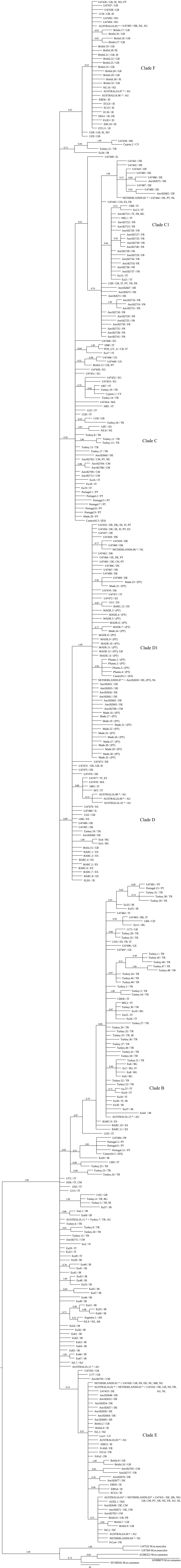
Clade F
Clade C1
Clade C
Clade D1
Clade D
Clade B
Clade E

REFERENCES (Figure S1)

Förster, D. W., Gündüz, İ., Nunes, A. C., Gabriel, S., Ramalhinho, M. G., Mathias, M. L., Britton-Davidian, J. & Searle, J. B. 2009 Molecular insights into the colonization and chromosomal diversification of Madeiran house mice. *Mol. Ecol.* **18**, 4477-4494.

Gündüz İ., Tez C., Malikov V., Vaziri A., Polyakov A. V. & Searle J. B. 2000 Mitochondrial DNA and chromosomal studies of wild mice (*Mus*) from Turkey and Iran. *Heredity* **84**, 458–467.

Gündüz İ., Auffray J.-C., Britton-Davidian J., Catalan J., Ganem G., Ramalhinho M. G., Mathias M. L. & Searle J. B. 2001 Molecular studies on the colonization of the Madeiran archipelago by house mice. *Mol. Ecol.* **10**, 2023–2029.

Gündüz İ., Rambau R. V., Tez C. & Searle J. B. 2005 Mitochondrial DNA variation in the western house mouse (*Mus musculus domesticus*) close to its site of origin: studies in Turkey. *Biol. J. Linn. Soc.* **84**, 473–485.

Ihle, S., Ravaoarimanana, I., Thomas, M. & Tautz, D. 2006 An analysis of signatures of selective sweeps in natural populations of the house mouse. *Mol. Biol. Evol.* **23,** 790-797.

Jones, E. P., Jóhannesdóttir, F., Gündüz İ., Richards, M. B. & Searle, J. B. 2011 The expansion of the house mouse into North-western Europe. *J. Zool.* **283**, 257-268

Nachman, M. W., Boyer, S. N., Searle, J. B. & Aquadro, C. F. 1994 Mitochondrial DNA variation and the evolution of Robertsonian chromosomal races of house mice, *Mus domesticus*. *Genetics* **136**, 1105–1120.

Prager, E. M., Sage, R. D., Gyllensten, U., Thomas, W. K., Hübner, R., Jones, C. S., Noble, L., Searle, J. B. & Wilson, A. C. 1993 Mitochondrial DNA sequence diversity and the colonization of Scandinavia by house mice from East Holstein. *Biol. J. Linn. Soc.* **50,** 85-122.

Prager, E. M., Tichy, H. & Sage, R. D. 1996 Mitochondrial DNA sequence variation in the eastern house mouse, *Mus musculus*: comparison with other house mice and report of a 75-bp tandem repeat. *Genetics* **143,** 427-446.

Prager, E. M., Orrego, C., Sage, R. D. 1998 Genetic variation and phylogeography of central Asian and other house mice, including a major new mitochondrial lineage in Yemen. *Genetics* **150,** 835-861.

Rajabi-Maham, H., Orth, A. & Bonhomme, F. 2008 Phylogeography and postglacial expansion of *Mus musculus domesticus* inferred from mitochondrial DNA coalescent, from Iran to Europe. *Mol. Ecol.* **17,** 627-641.

Searle, J. B., Jones, C. S., Gündüz, İ., Scascitelli, M., Jones, E. P., Herman, J. S., Rambau, R. V., Noble, L. R., Berry, R. J., Giménez, M. D. & Jóhannesdóttir, F. 2009a Of mice and (Viking?) men: phylogeography of British and Irish house mice. *Proc. R. Soc.* B**276**, 201-207.

Searle, J. B., Jamieson, P. M., Gündüz, İ., Stevens, M. I., Jones, E. P., Gemmill, C. E. C. & King, C. M. 2009b The diverse origins of New Zealand house mice. *Proc. R. Soc.* B**276**, 209-217.
