# Supplementary material for: Of Mice and ‘Convicts’: Origin of the Australian House Mouse, Mus musculus
Source: PLoS One. 2011 Dec 12;6(12):e28622. doi: 10.1371/journal.pone.0028622 (PMC3236204; doi:10.1371/journal.pone.0028622)
Supplement: Table S1 — Details of all house mice obtained from Australia and the Netherlands subject to D-loop sequencing. Geographical coordinates are represented as decimal degrees. Most Australian samples were provided by Museum collections and the remaining samples belong to private collections of Michael Nachman, Kristin Ardlie [Ardlie KG, Silver LM (1998) Low frequency of t haplotypes in natural populations of house mice (Mus musculus domesticus). Evolution, 52, 1185–1196] and Michael Driessen. Samples provided by Michael Driessen were collected during pest management work of the Resource Management and Conservation Division, Department of Primary Industries, and Water, Tasmania, following their standard ethical practice. All Dutch samples were provided by Adri Rol at the Zoological Museum of Amsterdam. ‘Sample ID’ corresponds to the original Museum Catalogue Number. (PDF) [file pone.0028622.s002.pdf]

Table S1 Details of all house mice obtained from Australia and the Netherlands subject to D-loop sequencing. Geographical coordinates are represented as decimal degrees. Most Australian samples were provided by Museum collections and the remaining samples belong to private collections of Michael Nachman, Kristin Ardlie [Ardlie KG, Silver LM (1998) Low frequency of t haplotypes in natural populations of house mice (*Mus musculus domesticus*). Evolution, *52*, 1185-1196] and Michael Driessen. Samples provided by Michael Driessen were collected during pest management work of the Resource Management and Conservation Division, Department of Primary Industries, and Water, Tasmania, following their standard ethical practice. All Dutch samples were provided by Adri Rol at the Zoological Museum of Amsterdam. ‘Sample ID’ corresponds to the original Museum Catalogue Number.

Sample ID	Sample provider	Latitude	Longitude	Haplotype	Clade
**AUSTRALIA**					
**Western Australia**					
36261	WAM	-31,2300	116,3017	AUSTRALIA.01	Clade E
49891	WAM	-31,2300	116,3017	AUSTRALIA.01	Clade E
44110	WAM	-28,2000	123,6000	AUSTRALIA.02	Clade E
44124	WAM	-28,2000	123,6000	AUSTRALIA.07	Clade F
56193	WAM	-26,9397	120,5581	AUSTRALIA.01	Clade E
48520	WAM	-25,0972	118,0083	AUSTRALIA.01	Clade E
49678	WAM	-29,2822	117,5742	AUSTRALIA.01	Clade E
49687	WAM	-29,2819	117,6428	AUSTRALIA.05	Clade F
56476	WAM	-34,2214	116,3741	AUSTRALIA.01	Clade E
56486	WAM	-34,0878	116,3194	AUSTRALIA.13	Clade B
ABTC07343	SAM	-30,8579	128,1011	AUSTRALIA.05	Clade F
ABTC07352	SAM	-30,8579	128,1011	AUSTRALIA.05	Clade F
ABTC07414	SAM	-33,4623	123,8421	AUSTRALIA.05	Clade F
ABTC07415	SAM	-33,4623	123,8421	AUSTRALIA.05	Clade F
ABTC07417	SAM	-33,4623	123,8421	AUSTRALIA.05	Clade F
ABTC07421	SAM	-31,8341	128,1776	AUSTRALIA.05	Clade F
ABTC63123	SAM	-21,3031	118,8611	AUSTRALIA.01	Clade E
**Northern Territory**					
ABTC41764	SAM	-25,5900	129,2858	AUSTRALIA.05	Clade F
ABTC24032	SAM	-23,7354	133,3307	AUSTRALIA.05	Clade F
ABTC24033	SAM	-22,7354	133,3307	AUSTRALIA.05	Clade F
ABTC28266	SAM	-12,1920	136,7742	AUSTRALIA.03	Clade E
M30774	ANWC	-12,7617	133,1033	AUSTRALIA.06	Clade F
**South Australia**					
ABTC37735	SAM	-37,0342	139,5117	AUSTRALIA.05	Clade F
ABTC37731	SAM	-37,1336	139,4804	AUSTRALIA.05	Clade F
ABTC37481	SAM	-37,0812	140,4142	AUSTRALIA.05	Clade F
ABTC37501	SAM	-37,5336	140,2228	AUSTRALIA.05	Clade F
ABTC37430	SAM	-38,0315	140,5635	AUSTRALIA.01	Clade E
ABTC26669	SAM	-32,0842	134,4352	AUSTRALIA.05	Clade F
ABTC26670	SAM	-32,0751	134,3943	AUSTRALIA.05	Clade F
ABTC26679	SAM	-31,4619	133,2820	AUSTRALIA.05	Clade F
ABTC26682	SAM	-31,4742	133,2545	AUSTRALIA.05	Clade F
ABTC35543	SAM	-29,3615	135,4335	AUSTRALIA.05	Clade F
ABTC35714	SAM	-29,1721	135,1100	AUSTRALIA.05	Clade F
ABTC35426	SAM	-29,0324	136,1556	AUSTRALIA.02	Clade E
ABTC36313	SAM	-28,5703	136,4655	AUSTRALIA.05	Clade F
ABTC36796	SAM	-31,2517	140,1515	AUSTRALIA.05	Clade F
ABTC36799	SAM	-31,2617	140,2807	AUSTRALIA.05	Clade F
ABTC36797	SAM	-31,2438	140,1035	AUSTRALIA.05	Clade F
ABTC36633	SAM	-30,2557	140,5826	AUSTRALIA.05	Clade F

Sample ID	Sample provider	Latitude	Longitude	Haplotype	Clade
**South Australia (cont.)**					
ABTC79657	SAM	-34,6004	138,7491	AUSTRALIA.11	Basal
ABTC33350	SAM	-35,8389	137,1679	AUSTRALIA.11	Basal
ABTC33353	SAM	-35,8389	137,1679	AUSTRALIA.11	Basal
ABTC33534	SAM	-35,8389	137,1679	AUSTRALIA.11	Basal
ABTC33610	SAM	-35,8389	137,1679	AUSTRALIA.11	Basal
ABTC33650	SAM	-35,8389	137,1679	AUSTRALIA.11	Basal
**Queensland**					
KA107	MN/KA	-27,5000	150,5000	AUSTRALIA.08	Clade D
KA112	MN/KA	-27,5000	150,5000	AUSTRALIA.08	Clade D
KA124aust	MN/KA	-27,5000	150,5000	AUSTRALIA.10	Clade D
KA1aust	MN/KA	-27,5000	150,5000	AUSTRALIA.08	Clade D
KA29aust	MN/KA	-27,5000	150,5000	AUSTRALIA.09	Clade D
KA35aust9205	MN/KA	-27,5000	150,5000	AUSTRALIA.08	Clade D
KA41aust	MN/KA	-27,5000	150,5000	AUSTRALIA.08	Clade D
KA9	MN/KA	-27,5000	150,5000	AUSTRALIA.08	Clade D
ABTC13890	SAM	-25,5023	138,4104	AUSTRALIA.02	Clade E
ABTC79716	SAM	-26,6422	149,6230	AUSTRALIA.02	Clade E
M30797	ANWC	-27,4900	152,8897	AUSTRALIA.08	Clade D
**New South Wales**					
KA35aust9204	MN/KA	-34,8058	145,8841	AUSTRALIA.04	Clade E
KA54	MN/KA	-34,8058	145,8841	AUSTRALIA.01	Clade E
M29998	ANWC	-35,5400	149,2100	AUSTRALIA.01	Clade E
**Australia Capital Territory**					
M30792	ANWC	-35,2192	149,1033	AUSTRALIA.01	Clade E
M30794	ANWC	-35,2192	149,1033	AUSTRALIA.01	Clade E
M30795	ANWC	-35,2192	149,1033	AUSTRALIA.01	Clade E
M30796	ANWC	-35,2192	149,1033	AUSTRALIA.01	Clade E
**Tasmania**					
A410	TMAG	-42,8817	147,3322	AUSTRALIA.01	Clade E
A504	TMAG	-42,9129	147,3545	AUSTRALIA.01	Clade E
A505	TMAG	-42,9129	147,3545	AUSTRALIA.01	Clade E
A1481a	TMAG	-42,8795	147,3275	AUSTRALIA.12	Basal
A1481b	TMAG	-42,8795	147,3275	AUSTRALIA.12	Basal
A1575	TMAG	-42,8731	147,3023	AUSTRALIA.01	Clade E
A3233	TMAG	-43,0624	147,8123	AUSTRALIA.01	Clade E
TAS1	MD	-42,9796	147,3262	AUSTRALIA.01	Clade E
TAS2	MD	-42,9796	147,3262	AUSTRALIA.01	Clade E
TAS3	MD	-42,9796	147,3262	AUSTRALIA.01	Clade E
TAS4	MD	-42,9796	147,3262	AUSTRALIA.01	Clade E
TAS5	MD	-42,9796	147,3262	AUSTRALIA.01	Clade E
TAS6	MD	-42,9796	147,3262	AUSTRALIA.01	Clade E
TAS7	MD	-42,9796	147,3262	AUSTRALIA.01	Clade E

Sample ID	Sample provider	Latitude	Longitude	Haplotype	Clade
**NETHERLANDS**					
**Rotterdam**					
5902	ZMA	51,9226	4,4707	NETHERLANDS.04	Clade E
5897	ZMA	51,9226	4,4707	NETHERLANDS.05	Clade C
5898	ZMA	51,9226	4,4707	NETHERLANDS.04	Clade E
**Oosthuizen**					
8139	ZMA	52,5747	4,9985	NETHERLANDS.06	Clade D
**Midsland noord**					
27479	ZMA	53,3631	5,2110	NETHERLANDS.05	Clade C
**Terschelling**					
27476	ZMA	53,3631	5,2110	NETHERLANDS.01	Clade E
27477	ZMA	53,3631	5,2110	NETHERLANDS.01	Clade E
**Montfort**					
4674	ZMA	51,1173	5,9521	NETHERLANDS.01	Clade E
**Maastricht**					
4393	ZMA	50,8498	5,6873	NETHERLANDS.07	Clade D
**Geulle**					
4391	ZMA	50,9225	5,7484	NETHERLANDS.03	Clade E
**Breda**					
5369	ZMA	51,5893	4,7745	NETHERLANDS.01	Clade E
5368	ZMA	51,5893	4,7745	NETHERLANDS.01	Clade E
**Groenekan**					
5949	ZMA	52,1229	5,1519	NETHERLANDS.02	Clade E
**Amsterdam**					
21355	ZMA	52,3739	4,8941	NETHERLANDS.01	Clade E
4401	ZMA	52,3739	4,8941	NETHERLANDS.05	Clade C
4669	ZMA	52,3739	4,8941	NETHERLANDS.05	Clade C
11604	ZMA	52,3739	4,8941	NETHERLANDS.03	Clade E

Sample providers

WAM – Western Australia Museum
SAM – South Australian Museum
ANWC – Australia National Wildlife Collection
TMAG – Tasmanian Museum and Art Gallery
ZMA – Zoological Museum of Amsterdam
MN/KA – Michael Nachman / Kristin Ardlie
MD – Michael Driessen
